# Hypoxia-responsive nanomaterials for tumor imaging and therapy

**DOI:** 10.3389/fonc.2022.1089446

**Published:** 2022-12-15

**Authors:** Yifei Xia, Shao Duan, Chaozhe Han, Chengwei Jing, Zunyu Xiao, Chao Li

**Affiliations:** ^1^ Department of Orthopedics, The Second Affiliated Hospital of Harbin Medical University, Harbin, China; ^2^ Department of Nuclear Medicine, The Fourth Hospital of Harbin Medical University, Harbin, China

**Keywords:** stimuli-responsive nanoparticle, hypoxia tumor microenvironment, drug delivery, cancer imaging, tumor microenvironment targeted theranostics

## Abstract

Hypoxia is an important component of tumor microenvironment and plays a pivotal role in cancer progression. With the distinctive physiochemical properties and biological effects, various nanoparticles targeting hypoxia had raised great interest in cancer imaging, drug delivery, and gene therapy during the last decade. In the current review, we provided a comprehensive view on the latest progress of novel stimuli-responsive nanomaterials targeting hypoxia-tumor microenvironment (TME), and their applications in cancer diagnosis and therapy. Future prospect and challenges of nanomaterials are also discussed.

## 1 Introduction

Hypoxia, caused by an imbalance in the supply and consumption of oxygen (O_2_) by rapidly proliferating tumor cells, is a hallmarker of numerous solid tumors (1, 2). The hypoxic TME can increase the generation of reactive oxygen species (ROS), which can disrupt normal tissues. It also disrupts cell cycle regulation and leads to treatment resistance, thereby contributing to cancer recurrence (3).

Overcoming hypoxia is a viable therapeutic strategy. Several techniques have been proposed and explored to cure hypoxia, including inhalation of hyperbaric oxygen, injection of erythropoietin, using vasodilators, or transfusing blood (4–7). Unfortunately, none of these tactics have been proven effective. Nanomaterials have brought unique insights into the therapy of tumor hypoxia in recent years, owing to the advancement of nanotechnology (8). Physical strategies and/or specific chemical have been used to enhance many basic types of nanomaterials, such as polymers (9, 10), liposomes (11), and inorganic nanoparticles (12). These nanomaterials can prevent tumor hypoxia in a variety of ways; for example, targeted transport or generation of oxygen, such as catalyzing the decomposition of higher concentrations of H_2_O_2_ in the microenvironment to oxygen or using perfluorocarbons with high oxygen affinity. Constructing hypoxia-activated chemical bond-modified nanostructures, such as the design of nitro-coupled polymeric drugs, which can disrupt the structure and release the drug by nitro cleavage (13). In addition, researchers have designed active targeting vectors or anaerobic bacterial vectors in combination with *in vitro* adjuvant therapy, subsequently activating the drugs by means of radiotherapy and photothermal therapy. On the other hand, nanomaterials could also have great potential to improve tumor diagnostic strategies. Current clinical diagnostic imaging techniques for tumors include computed tomography (CT)/magnetic resonance imaging (MRI), positron emission tomography (PET), and near-infrared fluorescence (NIRF) imaging (14). Given that traditional contrast agents often do not efficiently accumulate in hypoxic tumor regions, these imaging modalities have gradually failed to satisfy the demand for early and accurate diagnosis. Unlike conventional contrast agents or probes, nanomaterials could accumulate in TME through decomposition and self-assembly or targeting various components associated with hypoxia through modified ligand materials to achieve stable and highly specific imaging results (15).

In the present study, we focus on new advances in nanomaterials for cancer imaging and therapy. We first provided an overview of the physicochemical and biological aspects of hypoxia and then illustrate strategies and recent advances that have been used to develop hypoxic stimuli-responsive nanomaterials. The major limitations and future prospects for clinical translation are also discussed.

## 2 Characteristics of hypoxia microenvironment

**Figure 1:**
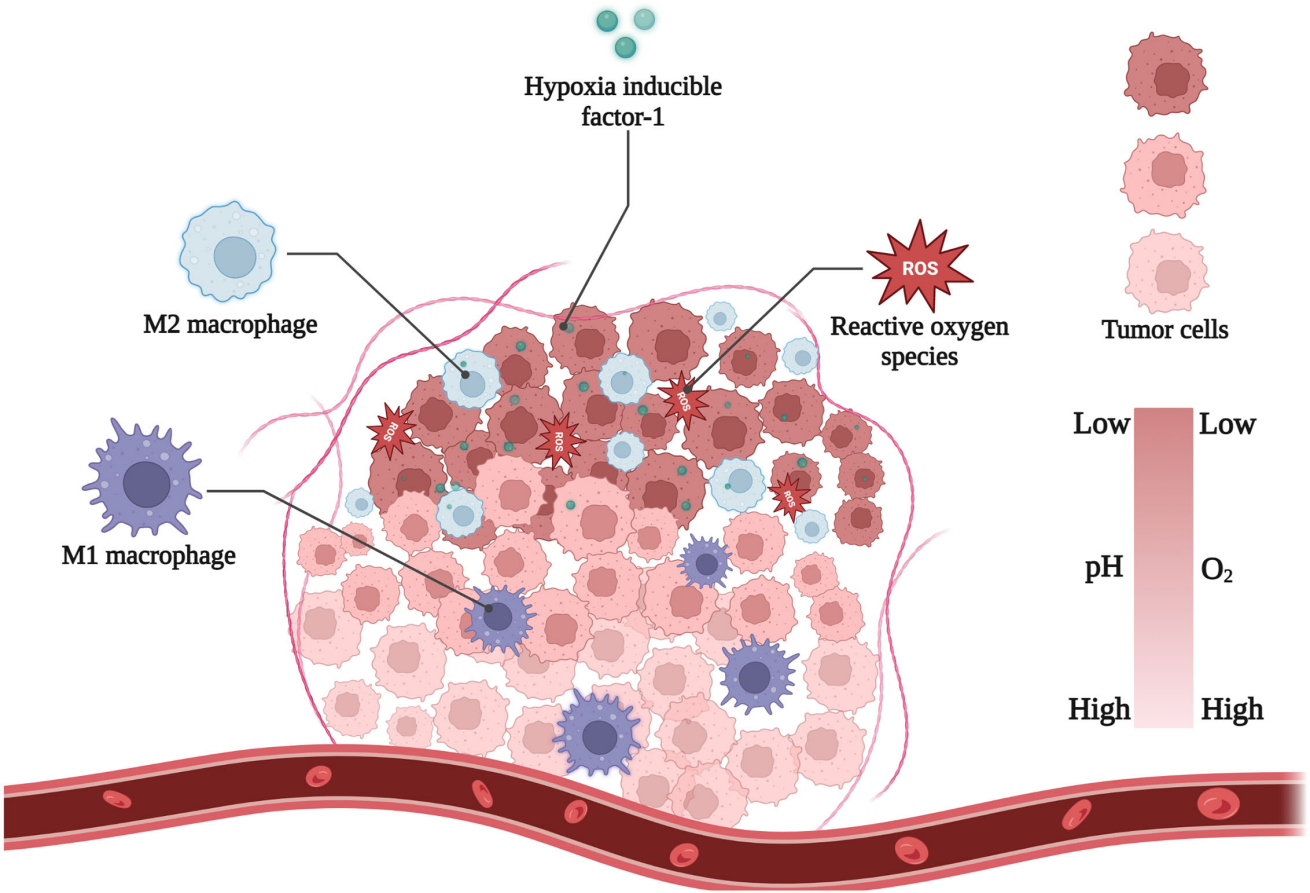
Main hallmarks of hypoxic TME. Figure created with Biorender.com.

Hypoxia is a hallmark of solid tumors. The oxygen tension in most normal tissues with abundant blood supply is approximately 30-70 mmHg. In contrast, the oxygen tension around most tumor cells varies from 2.5 mmHg to 7.5 mmHg (16). Hypoxic regions can accelerate the formation of the tumor barrier and increase cytokine secretion (6). These abnormal tissue conditions give tumor cells different physical, chemical, and biological characteristics, such as low pH and high redox potential. Understanding these characteristics is useful for researchers to improve the design of new nanomaterials and lead to more effective diagnosis and treatment (**Figure 1**).

### 2.1 Low pH

Tumor cells are usually in a low-oxygen environment owing to the insufficient blood supply. This environment exacerbates anaerobic glycolysis of tumor cells, which produces large amounts of lactic acid, protons, and carbonic dioxide into the TME during tumor expansion (17). Tumor cells catalyze the export of these acidic metabolites by regulating transmembrane ion fluxes. However, the hypoxic TME is distant from the blood vessels, the clearance of acidic metabolic waste is obstructed (18). Thus, these effects endow the hypoxic tumor region with a relatively low pH around 6.2–7.2 (19). Furthermore, in order to exacerbate the migration and invasion of hypoxic tumor cells, low pH environment often facilitates the destruction of the extracellular matrix (20).

### 2.2 High redox potential

High redox potential is another specific feature found in hypoxic TME (21). Normal cells tend to maintain a dynamic redox balance, but tumor cells generally exhibit a state of oxidative stress and generate large amounts of ROS—100 times higher than that in normal cells—to adapt to hypoxia and glucose deprivation (22). ROS are important components of redox reactions, resulting in tissue damage and stimulation of metastasis-associated growth factor (23–25). To avoid redox imbalances and prevent fatal levels of ROS, tumor cells’ antioxidant systems become more activated than those of cells under normoxic conditions. For examples, the activities of reductase systems, including azo reductase, nitro reductase (NTR), and NAD(P)H, are enhanced, and the amounts of antioxidant agents such as cysteine and glutathione, are increased (26). These properties play a crucial role in the formation of hypoxia-induced chemical bonds that react with different types of reductases, including nitroimidazole, azo, and others.

### 2.3 Tumor-associated macrophages

Tumor-associated macrophages (TAMs) constitute a significant percentage of TME, accounting for up to 50% of solid tumors (27). It is well known that tumor cells secrete a variety of chemokines, such as CCL2, CXCL1, CXCL8, etc (28–30). Macrophages in the blood are attracted by these cytokines, accumulate near the TME, and develop into TAMs. Subsequently, hypoxic tumor regions produce hypoxia-inducible factor-1 (HIF-1)-dependent cytokines (CXCL12, VEGF, and CXCL4), causing TAMs to accumulate in the avascular zone (31). TAMs are highly associated with tumor progression and poor prognosis. TAMs highly express IL-6, CXCL-8, and IL-10, which promote tumor cell growth, suppress the immune response of cytotoxic T cells and reduce the effects of chemotherapy (32, 33). TAMs also secrete matrix metalloproteinases (MMPs), histone proteases, and serine proteases, which could diminish the connections between the endothelial basal lamina and endothelial cells, as well as accelerate tumor cell migration (34).

### 2.4 Hypoxia-inducible factor-1

The hypoxic cellular response is primarily driven by hypoxia-inducible factor-1 (HIF-1). Hypoxia-inducible factor-1 is considered as a major transcriptional regulator to hypoxia in a variety of cells, which is composed of HIF-1α and HIF-1β subunits (35). In normal tissues, HIF-1α is hydroxylated by oxygen, attaches to ubiquitin ligase, and is subsequently destroyed by proteasomes. Under hypoxic conditions, HIF-1α hydroxylation is blocked, resulting in its binding to HIF-1β and translocation to the nucleus (36). To reduce the negative effects of hypoxia, HIF-1 can activate genes that regulate glucose transporters and glycolytic enzymes, in addition to switching tumor cells from aerobic respiration to anaerobic glycolysis (37). HIF-1 can stimulate the hepatocyte growth factor (HGF)/HGF receptor (c-MET) signaling pathway (38), boosting tumor cell invasion and metastasis (39–41). Therefore, HIF-1 is frequently used to predict poor tumor prognosis.

## 3 Strategies for overcoming hypoxia

### 3.1 Active targeting nanomaterials

#### 3.1.1 Obligate anaerobes

The ability of bacteria to treat cancer was first discovered by Dr. William B. Coley in the early 19th century. He established a new approach for cancer therapy, namely anaerobic targeted therapy (42). The principle of anaerobic treatment of tumors is that the anaerobes could proliferate after entering into tumors, due to the anoxic environment. By depleting the nutrients needed for tumor growth, bacteria could kill tumors. However, early research was unsuccessful, especially for large solid tumors (≥500 mm^3^ in volume). The reason is that anaerobes could selectively proliferate and destroy hypoxic tumor regions but leave a well-oxygenated outer rim of the large solid tumors that can lead to tumor recurrence (43–45).

**Figure 2:**
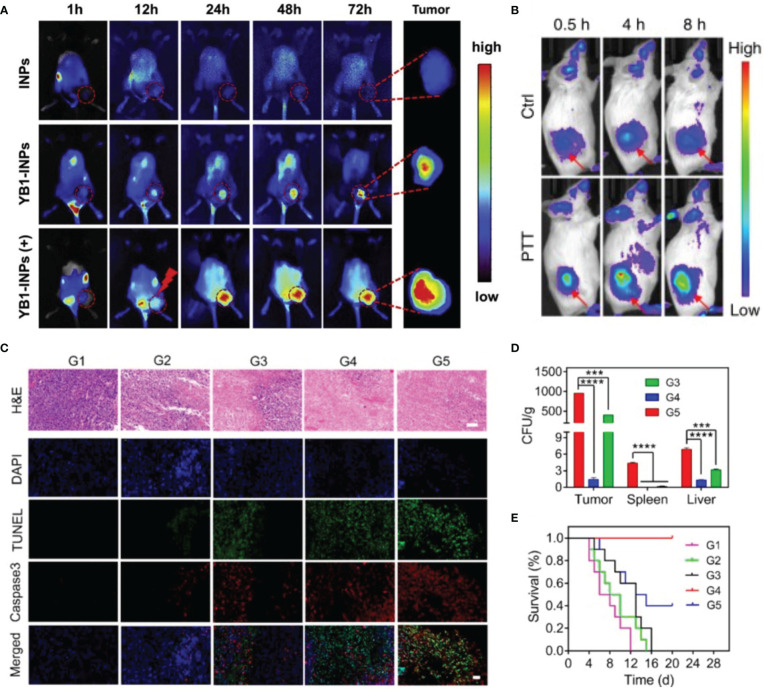
Bacteria-mediated nanoparticles have efficient imaging properties and superior therapeutic outcome. **(A)** *In vivo* FL imaging of YB1-INPs, (+) refers to laser irradiation at 12 h. Reprinted with permission (46). Copyright 2019 Biomaterials. **(B)** *In vivo* FL imaging of OMV-labeled SP-AgNPs. Reprinted with permission (48). Copyright 2020 Nature Communications. **(C-E)** Therapeutic efficacy and safety evaluation of OMV-NPNs@Pt. Reprinted with permission (47). Copyright 2021 Nano Letters. **(C)** H&E, TUNEL, and Caspase 3 staining of tumor sections after different treatments. Scale bar = 50 μm. **(D)** The bacteria distribution in vital organs after different treatments **(E)** Survival rate of the mice with various treatments. ***P < 0.001, ****P < 0.0001.

Bacteria-mediated hypoxia-specific nanoparticles have demonstrated therapeutic efficacy. Nanomaterials could help anaerobes cross physiological boundaries to improve their anticancer activity (**Table 1**). There are three types of nanoparticles: bacterial complexes with nanomaterials, anaerobic bacterial spore germination marker-targeting nanomaterials, and bacterial secretions coupled to nanomaterials (58) (**Figure 2**). The coupling of nanocarriers with strains is a typical building method. Salmonella Typhimurium YB1 (YB1) is a typical biologically modified bacterium that can easily form amide bonds with micro photosensitizers (INPS) (59). Zheng et al. created a biological/abiotic nanocomposite (YB1-INPS) that retained both YB1 activity and the photothermal efficacy of INPS. YB1-INPS had an excellent fluorescence imaging ability, which clearly revealed the tumor area. After exposure to the tumor, NIR light activates the photosensitizers, which can destroy tumors and the leftover bacteria (46). Furthermore, with the specific germination of Clostridium difficile spores under anoxic conditions, researchers can leverage this trait to sequentially introduce spores and specific antibody-nanoconjugates into the body; the antibodies subsequently signal spore germination to detect the tumor site (13). Rare earth upconversion luminescent nanomaterials (UCNR) or Au nanorods can be used in nanomaterials to realize the integration of NIR imaging and photothermal therapy (58). This antibody-targeted diagnosis and treatment can enhance the imaging contrast, prolong the cycling time, and improve the therapeutic effect on tumors.

**Table 1:** Active targeting nanomaterials and nanoparticles for oxygen transport.

Name/Target (Year)	Materials	Drug	Size (nm)	Zeta (mV)	Tumor model	Imaging mode	Results
**Obligate Anaerobes**							
**1. YB1-INPS**	PLGA	INPS	≈1000	−	MB49 cells/ C57BL/6 mice	NIR fluorescence imaging	Highly selective hypoxia-targeting of delivering INPs. (46)
**-2019**							
**2. SP-AgNPs**	AgNPs	——	15	-2	B16F10 cells/ BALB/c mice	Bioluminescence imaging	Improve tumor therapy biosafety via neutrophil infiltration. (47)
**-2021**							
**3. OMV-NPNs@Pt**	PEG-b-PLGA	Cisplatin	149.0 ± 3.1	-4.16 ± 0.32	EMT6/CT26 cells/	——	Excellent tumor targetability and complete eradication of tumors through PTT combination therapy. (48)
**-2020**					BALB/c mice/ C57BL/6 mice		
**Targeted HIF-1**							
**4. DG-PEG-LA-Lys-9R**	2-DG-PEG	siRNA	218.39 ± 9.00	−0.01 ± 0.07	HepG2, U87MG, SGC-7901, MCF7 cells/ BALB/c nude mice	NIR fluorescence imaging	Enhance antitumor efficacy and reduce organ toxicity. (49)
**-2015**							
**5. Gd@C82(OH)22**	Gadolinium metallofullerene	——	40.5~175.7	——	MDA-MB-231, BT-549 cells/	——	As a non-toxic inhibitor of HIF-1a and TGF-b activities, efficient elimination of breast cancer stem cell. (50)
**-2015**			(pH 4.3~7.4)		Female BALB/c nude mice		
**Targeted TAM**							
**6. LCL-PLP**	LCL	Prednisolone phosphate	≈100	——	B16.F10 cells/ Male BALB/c nude mice	——	Reduction of the TAM-mediated production of pro-angiogenic factors. (51)
**-2008**							
**7. CaBP(^99m^Tc)-PEG**	CaBP-PEG	^32^P	≈40	-0.5	4T1, CT26 cells/ Female BALB/c mice	SPECT imaging	Great biocompatibility and prolonged biodistribution. (52)
**-2018**							
**8. MPEI/pCAR-IFN-γ**	MPEI	IFN-γ	32.8 ± 2.1	3.2 ± 1.7	Neuro-2a mouse neuroblastoma cells/ female A/J mice	NIR fluorescence imaging	Effectively detected TAM biomarker and improve anti-tumor immunity. (53)
**-2021**							
**Oxygen Transport**							
**9. PFTBA@HSA**	Perfluorocarbon	O_2_	150	-35	CT26, SUM149PT cells/ Male	——	Increase RBCs infiltration and O2 delivery via physically dissolved oxygen, reverse tumor resistance to radiotherapy. (54)
**-2018**					BALB/c mice		
**10．PFC@PLGA-RBCM**	(PFC)	O_2_	290	-10.8	4T1, CT26 cells/ Female nude mice	——	Prolonged blood circulation and enhance radiotherapy. (55)
**-2017**	PFC-PLGA						
**11. IR780@O2-FHMON**	FHMON	O_2_	180	-26	Panc-1 cells/ nude mice	Ultrasound Molecular Imaging	High storage capacity and binding sites, mitigate hypoxia tumor induced resistance. (56)
**-2017**							
**12. Gd@HbCe6-PEG**	Gd-based nanostructures	Hb	21	——	4T1 cells/ BALB/C mice	MR imaging	Great biocompatible and non-toxic, enabled tumor-specific PDT by ameliorating tumor hypoxia. (57)
**-2020**							

SPECT imaging, single-photon emission computed tomography imaging.

Gram-negative bacteria secrete outer membrane vesicles (OMVs) under certain conditions (60). The surface of OMVs contains bacterial antigens, moreover, OMVs have the advantages of good biocompatibility, safety, and modifiability (61). It is known that neutrophils may detect and ingest pathogens by identifying pathogen-associated molecular patterns (PAMPs) (62). By formulating the use of pathogenmimicking nano-pathogenoids (NPNs) to attract circulating neutrophils, researchers could produce a nano-sized replica of the original bacteria with similar pathological activities by covering NPs with OMVs (63). Thus, researchers proposed a combination strategy by leveraging this property: first, inject salmonellas into the body and allow the bacteria to infiltrate the tumor and recruit neutrophils; next, inject sialic acid (SA)-modified silver nanoparticles (AgNPs) *in vivo*, which can target the TME by recognizing neutrophil L-selectin (47). In addition, by combining OMVs with cisplatin-loaded nanoparticles, Wang et al. developed nano-bionic pathogens (NPNs@Pt). These pathogens could target hypoxic tumor areas and activate inflammatory responses after photothermal therapy (PTT), leading to massive neutrophil infiltration. The neutrophils rapidly break down OMVs and release cisplatin to kill tumor cells—within four hours. This strategy was highly effective in mice, completely curing them after two treatment sessions (48).

#### 3.1.2 HIF-1

Following the finding that HIF-1 may be used as a tumor therapeutic target, various small molecule inhibitors, medications and siRNAs have been developed (64, 65). By encouraging HIF-1 protein degradation or by preventing HIF-1 mRNA production, several small inhibitors have demonstrated a high rate of HIF-1 activity inhibition (66, 67). However, these small inhibitors have a relatively high risk of clinical failure, which may be attributed to the high redundancy and complexity of the TME. siRNAs prevent tumor growth by blocking HIF-1 transcription and translation. However, they are easily degraded by various nucleases in the circulation (68). Recent studies have revealed that some indirect methods, such as nanomedicines, may be a strong strategy to translate HIF-1 directed therapies to clinical development (**Table 1**). By loading inhibitors into NPs, the complexes could easily target HIF-1α, avoiding drug degradation (69, 70). Zhu et al. created a functional nanocarrier using 2-deoxyglucose (DG)-polyethylene glycol (PEG) and fluorescent CdTe quantum dots (Qds). When the nanocarrier reached the hypoxic region, it self-ruptured and released siRNA, which could target and silence tumor cells, whereas fluorescent Qds could actively monitor the transport process (49). Another composite nanomaterial, Gd-metallofullerenol nanomaterial (Gd@C82(OH)22)—a dual-action inhibitor of HIF-1α and TGF-β—showed excellent targeting ability and inhibition in a triple-negative breast cancer (TNBC) mouse model. This nanomaterial is non-toxic in normal tissues, but the particle size is reduced in the TME to penetrate the tumor center and significantly inhibit tumor growth (50).

#### 3.1.3 TAMs

Tumor-associated macrophages (TAMs) are important components of immune cells present in high numbers in TME. Current nanoparticles targeting TAMs are mainly for inhibiting their expression or deplete their number (**Table 1**). For example, encapsulating glucocorticoids (such as prednisolone) with long-circulating liposomes (LCLs) can passively target tumors *via* the enhanced permeability and retention effect (EPR); i.e., gradually releasing encapsulated hormones and blocking monocyte differentiation, thereby effectively preventing TAM production (51). Tian et al. combined calcium bisphosphate with ^99m^TC/^32^P-labeled PEG, which can deplete TAM and promote the normalization of tumor blood vessels, laying a solid foundation for subsequent radioimmunotherapy (52). On the other hand, by encoding specific plasmid DNA, M2 macrophages can be transformed into M1 macrophages *via* the NF-κB and STAT pathways. A recent study developed a PEI-encapsulating mannose nanocomplex (MPEI), which could target mannose receptors overexpressed on the surface of TAMs and transfect plasmids into TAMs (53). Meanwhile, combining IL-12-overexpressing plasmids with vincristine-containing nanocarriers permitted circulation *in vivo* and long-term uptake by TAMs, even up to seven days. Thus, a microscopic reversal of many M2 macrophages to the M1 phenotype was observed (71).

### 3.2 Relieve hypoxia

#### 3.2.1 Hypoxia-triggered oxygen transport

In the last century, hyperbaric oxygen (HBO) therapy had been shown to enhance the sensitivity of cancer cells to radiotherapy and chemotherapy; thus, doctors have applied it to cancer patients as an adjuvant (72). However, side effects, such as barotrauma and hyperoxic seizures, limit HBO’s clinical application (73, 74). Recently, certain inorganic nanomaterials, such as perfluorinated carbons and carbon nanotubes, have shown efficient oxygen-carrying capacities (75) (**Table 1**). One study combined albumin with perfluorotributylamine, and designed a two-step oxygen delivery system (PFTBA@HAS). First, oxygen is released through the passive targeting of nanoparticles, followed by the platelet inhibitory effect of PFTBA, which inhibits aberrant tumor angiogenesis, thereby facilitating secondary oxygen release (54). Liu et al. loaded perfluorinated carbons (PFC) in poly (lactic-co-glycolic acid) (PLGA) and encapsulated them in red blood cell membranes. This particle exhibited a significant oxygen-carrying capacity and an extremely long blood circulation time (55). Unfortunately, the dissolved oxygen in perfluorocarbons can only be released by simple diffusion, thus lead to low oxygen release rate. Therefore, researchers could utilize specific nanocarriers by external stimulation, promoting the release of oxygen more quickly and effectively. Chen et al. improved the oxygen-releasing nanoplatform by designing fluorocarbon chain-functionalized hollow mesoporous organosilica nanoparticles (FHMONs), which have sufficient storage capacity for acoustic sensitizers (IR780) and oxygen. Ultrasonography could release large amounts of oxygen by triggering the carrier to decompose, as well as generating ROS that kill tumor tissue (56).

Despite the high oxygen carrying capacity, these nanoparticles have a poor histocompatibility. Hemoglobin (Hb) has received increasing interest as a high-oxygen transporter with excellent biocompatibility (76–78). A synthesized paramagnetic nanoprobe (Gd@HbCe6-PEG) was reported to enhance the therapeutic effect of photodynamic therapy (PDT) by retaining the oxygen-carrying ability of Hb. The fluorescence imaging demonstrate that this strategy can significantly alleviate the hypoxic condition (57).

#### 3.2.2 Hypoxia-triggered oxygen production

**Figure 3:**
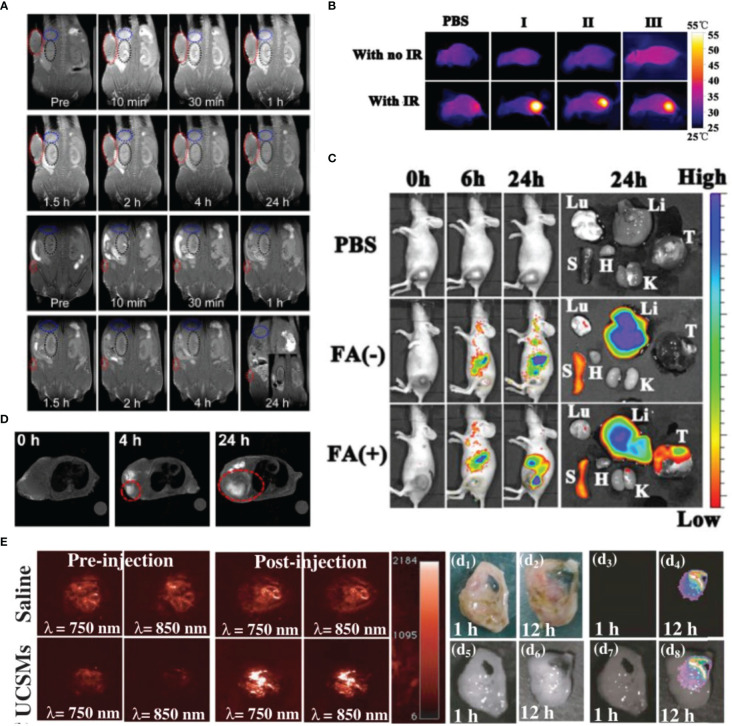
Variety of metal nanoparticles have great imaging effect. **(A)** *In vivo* MRI images of the Fe_2_O_3_ SPs, the tumor sizes are about 300 and 5 mm^3^, respectively. Reprinted with permission (83). Copyright 2020 ACS Nano. **(B, C)** Reprinted with permission (84). Copyright 2021 Biomaterials. **(B)** The photothermal images of tumor-bearing mice with injection of Hb-PDA-Fe@GOD@PEG-FA after being exposed to 808 nm irradiation. **(C)** *In vivo* biodistribution of Hb-PDA-Fe@GOD@PEG-FA nanoparticles. **(D)** *In vivo* MRI of tumor-bearing mice before and after intravenous injection of mCMSNs. Reprinted with permission (82). Copyright 2019 ACS Nano. **(E)** Representative 2D photoacoustic images of solid tumors before and after injection of saline/MnO_2_-UCSMs. Reprinted with permission (81). Copyright 2015 Advanced Materials.

Owing to the high redox potential in the tumor regions, large amounts of H_2_O_2_ and ROS cannot be decomposed. In the acidic environment of the hypoxia TME, metal nanoparticles can be activated to decompose H_2_O_2_ to oxygen and hydroxyl radicals (79, 80). In this section, we review metal nanoparticles based on their unique catalytic capacity (**Table 2**, **Figure 3**).

**Table 2:** Hypoxia-sensitive nanoparticles for oxygen production and hypoxia-responsive chemical bones nanoplatforms.

Name/Target (Year)	Materials	Drug	Size (nm)	Zeta (mV)	Tumor model	Imaging mode	Results
**Oxygen Production**							
**13. MnO_2_-UCSMs** **(2015)**	MnO_2_, UCSM	Mn^2+^	——	——	4T1 cells/ Female BALB/C mice	UCL imaging	Simultaneous diagnosis and positioned treatment of tumors via the radio/photodynamic therapy. (81)
**14. mCMSNs** **(2019)**	DPSNs	Cu^2+,^ Mn^2+^	130	-10	MCF-7, A549 and NHDF cells/ Female BALB/C mice	MR imaging	Monitor and enhance the synergistic CDT/PDT anticancer treatment. (82)
**15. Fe_2_O_3_ SPs** **(2020)**	Fe_2_O_3_	Fe^2+^	15	-17.5	4T1 cells/ Female BALB/c nude mice and female BALB/c mice	MR imaging	High signal-to-noise ratio resulting in excellent MR imaging capacity. Great biocompatibility, easy clearance. (83)
**16. Hb-PDA-Fe@GOD** **@PEG-FA (2021)**	Hb-PDA NPs	PDA, GOD Fe^2+^	200	-17	B16.F10 cells/ Male BALB/c nude mice	NIR fluorescence imaging	Manipulates the TME as needed to indicate synergistic therapy. (84)
**Chemical bonds**							
**17. HRNP/ siRNA** **(2020)**	Cationic lipid-like compound	CDC20 siRNA	54.7	——	MCF-7, Luc-HeLa cells/ Female BALB/c nude mice	NIR fluorescence imaging	Sufficiently silencing of CDC20 expression, exhibited potent antitumor efficacy. (85)
**18. HA-Fe-NIs-DOX** **(2018)**	Ferrocene-based redox polymers	DOX	83.03 ± 1.29	-41.3	PC3, DU145 and 293T cells/ Male BALB/c nude mice	NIR fluorescence imaging	Improved synergistic mechanisms of antitumor agents and chemo-/radiotherapy by effective DOX release. (86)
**19. UIO-NBD** **(2021)**	Iron oxide	——	10.06	-40.4	MDA-231, 4T1, MCF-7, B16 cells/ Female BALB/c mice	MR imaging/ NIR fluorescence imaging	Notable efficiency of penetration and accumulation inside tumors resulting in dual-mode imaging. (87)
**20. AQ4N-Cu(II)-** **Apt_Ce6_-GNPs (2017)**	Monodispersed gold nanoparticle	Ce6, AQ4	137.07 ± 4	-6.1 ± 0.9	HepG2, LO2, HeLa cells/ BALB/c nude mice	NIR fluorescence imaging	Enhanced tumor specificity and PDT/PTT/chemotherapy functions. (88)
**21. PEG-PO-PCL-PO** **-PEG (2019)**	PEG, PCL	GOD, AQ4	180	——	Hep3B cells/ Nude mice	NIR fluorescence imaging	Synergistic effects of starvation therapy and chemotherapy via a programmable self-destruction. (89)
**22. HCHOA** **(2019)**	HAS	Oxaliplatin	100~150	——	4T1 cells/ Female BALB/c nude mice	NIR fluorescence imaging	Strong imaging, deep penetration of hypoxia TME resulting in effective combined therapy. (90)
**23. AMOFs** **(2019)**	Metal−organic frameworks	siRNA, DOX	152.4 ± 6.1	23.1±1.8	MCF-7 cells/ Female BALB/c nude mice	NIR fluorescence imaging	Efficiently break hypoxia-induced chemoresistance via inhibiting the expressions of HIF-1α. (91)

DPSNs, dendritic mesoporous silica nanoparticles.

Hb-PDA NPs, hemoglobin conjugated polydopamine nanoparticles.

##### 3.2.2.1 Manganese

Manganese (Mn) is an element necessary for human metabolism, with low toxicity and high biocompatibility. Owing to their unique hollow structure and chemical properties, Mn-based nanoplatforms had demonstrated promising results in bioimaging and tumor-targeted therapy (92–94). Mn_x_O_y_, such as MnO_2_, can degrade and release Mn^2+^ into tumor regions. Manganese ions converts endogenous H_2_O_2_ into highly toxic hydroxyl radicals (-OH) through a Fenton-like reaction. Hydroxyl radicals aggravates the state of cellular oxidative stress, thereby realizing chemodynamic therapy (95). MnO_2_ nanoparticles can also enhance reoxygenation in tumors and destroy the hypoxic TME, thus enhancing the therapeutic effect of radiotherapy. This combination therapy can prolong the survival of breast cancermouse models by three to five times (96). At the same time, MnO_2_ can react with H^+^ and GSH in the TME, leading to the increase of ROS levels to promote tumor cell apoptosis. Moreover, the released Mn^2+^ can simultaneously as a contrast agent (CA) for T1-weighted MR imaging (97, 98). Although the T1-MRI performance of MnO_2_ nanoparticles is not as good as that of commercial Gd chelates, the loaded drugs can be released to perform a variety of treatments under the guidance of MR imaging (99). Wenbo et al. developed intelligent nanomaterials based on the MnO_2_ nanosheets anchored with upconversion nanoprobes (UCSMs). Under the influence of acidic tumor pH levels, the outermost MnO_2_ sheet disintegrates to expose the responsive luminescence signal of the inner layer, allowing physicians to achieve synergistic oxygen uplift guided by high-resolution upconversion luminescent (UCL) imaging (81). Another strategy is to combine metal ions with MnO_2_, such as Au or Cu ions, to enhance the efficacy of chemodynamic therapy/radiotherapy. Liu et al. wrapped the cancer cell membrane on the surface of mesoporous copper/manganese silicate nanospheres (mCMSNs) and delivered it to the tumor area accurately using the adsorption of the same cell membrane. They combined active targeting, PDT, Fenton-like reactions, MRI, and oxygenation, which provided an excellent idea for the innovation of metal nanoparticles (82).

##### 3.2.2.2 Fe

In addition to Mn-based nanomaterials, Fe-based nanoplatforms could also achieve the integration of treatment and imaging by releasing Fe^2+^ (100). Existing clinical CAs are mainly gadolinium (Gd) chelates, which have short relaxation times and nephrotoxicity (101, 102). Superparamagnetic iron oxide nanoparticles (SPIONPs) have been commercialized as a type of contrast agent for MRI, but their clinical application is limited owing to their poor T2-weighted imaging. To improve the imaging capability of SPIONPs, one strategy is to develop quasi-amorphous and hierarchical Fe_2_O_3_ supraparticles. Compared to ordinary SPIONPs, Fe_2_O_3_ supraparticles have higher degradation-induced imaging signals. This self-degradation ability also reduces the metabolic burden on the kidneys and avoids side effects similar to those of Gd contrast agents (83). Furthermore, single-atom catalysts are a viable strategy for enhancing the Fe-based nanoplatforms therapeutic capabilities of tumors. Chen et al. fabricated single-atom Fe nanocatalysts (SAF NCs) with single-atom Fe being isolated in nitrogendoped carbon. Fe atom could catalyze the Fenton reaction under acidic TME to release -OH, which can cause ferroptosis by massive induction of lipid peroxides. At the same time, based on the photothermal performance of the amorphous carbon, mild-photothermal augmented Fenton catalytic therapeutics could complete eliminate tumors (103). In addition, the Fenton reaction consumes H_2_O_2_ in the tumor area and causes the irreversible transformation of Fe^2+^ into inactive Fe^3+^, eventually leading to the failure of antitumor therapy. Therefore, it is important to ensure the continuous generation of Fe^2+^ and H_2_O_2_. Yuan et al. developed a multi-layer iron-based nanomaterial consisting of Hb, Fe^3+^, a dopamine core, a glucose oxidase interlayer, and a folic acid-modified polyethylene glycol (PEG-FA) corona. The PEG-FA corona is considered asa tumor-targeting agent, which could also protect Hb and glucose oxidase from proteases in circulation. After reaching the hypoxic TME, the nanomaterial decomposes and releases polydopamine, which is employed to increase the local temperature under NIR irradiation. Hb supplies oxygen to promote glucose oxidase activity and achieve rapid glucose consumption and H_2_O_2_ formation. Polydopamine can also continuously reduce Fe^3+^ to Fe^2+^, which further catalyzes the conversion of H_2_O_2_ to -OH *via* the Fenton reaction (84). Finally, this nanomaterial achieved photothermal-starvation-chemodynamic therapy for effective tumor treatment.

### 3.3 Hypoxia-triggered chemical bonds

Hypoxia-activated prodrugs are a class of inactive prodrugs that require enzymatic activation (by electron oxidoreductases) to produce cytotoxic substances (104, 105). The unique properties of hypoxia-activated prodrugs are derived from hypoxia-responsive chemical bonds, including nitro, azo, and AQ4N bonds. The variety of such chemical bonds under hypoxia endows nanomaterials with diverse functions that enhance their therapeutic and diagnostic effects. In this section, we discussed recent advances of hypoxia-responsive chemical bonds nanoplatforms (**Table 2**).

#### 3.3.1 Nitroimidazole

Since the 1970s, nitroimidazoles have been widely used in MRI, PET, fluorescence imaging, radiotherapy, responsive prodrugs, and other fields (106). Nitroimidazole compounds can be used as imaging agents and prodrugs because the nitro group (RNO^2-^) can be reduced under nitroreductase to generate free radical anions (RNO^2-^) (107). In normal tissues, this process is reversible, whereas in hypoxic tumor cells, products are further reduced to hydroxylamine (RNHOH) or amine (RNH2), both of which bind to proteins and are trapped in tumor cells. 2-Nitroimidazole (NI), one of the most commonly used nitroimidazole compounds, can impart hypoxic responsiveness to various nanomaterials (108).

In siRNA therapy, researchers prefer to develop highly stable liposomes, such as methoxy- polyethylene glycol (mPEG) or alkylated PEI (109, 110). However, stable liposomes can also hinder the release of siRNAs and reduce the efficiency of gene silencing. The hypoxia-responsive nanoparticle (HRNP) nanoplatform—composed of the 2-nitroimidazole-L-glutamine polymer and methoxy polyethylene glycol—solved this problem (85). HRNP exhibited prolonged blood circulation and high tumor accumulation, as well as delivered an siRNA silencing efficiency of more than 90%. Gao et al. successfully modified branched polyethyleneimine with alkylated NI (C6-NI), which could effectively condense siRNA to form a hypoxia-responsive polyethyleneimine carriers. This nanocarrier can be self-assembled into micellar polymers under physiological conditions for improved stability. After being transported into the hypoxic tumor cells, the structure of micellar polymers would be loosened by reduction of NI to facilitate the siRNA dissociation in the cytoplasm (111). Furthermore, NI can be combined with Fe-based nanomaterials, which can act as a sensitizer for radiotherapy. By examining enhanced radiotherapy, Mao et al. combined ferrocene with NI and modified with hyaluronic acid (HA) to synthesize HA-Fe-NIS nano-micelles. Under hypoxic conditions, HA-Fe-NIS could completely release the loaded doxorubicin within six hours, and this smart design enhanced the tumor fluorescence imaging intensity. Most importantly, compared to HA-Fe micelles, tumors in the HA-Fe-NIS group showed more obvious DNA damage after radiotherapy treatment, proving that HA-Fe-NIS had a strong radio sensitizing effect on hypoxic tumor cells and had clinical application value (86). NI derivatives and cysteine-modified ultrasmall iron oxide nanoparticles (UIOs) have excellent physical and chemical properties. UIOs have a very small particle size, allowing them to easily penetrate endothelial cells to reach the TME. Simultaneously, the modified nitroimidazole group can induce covalent cross-linking of UIOs under hypoxia to increase their particle size and promote accumulation and retention time in the hypoxic region. By measuring nano-aqueous solution under different oxygen conditions, the relaxation value of UIOs increased from 12.8s^-1^ to 21.4s^-1^ under hypoxia, indicating increased water proton transverse relaxation and contributing to enhanced T2-weighted MRI. UIOs and assembly-responding fluorescence dyes (NBD) can also provide dual-mode (MRI/fluorescence imaging) imaging *in vivo* (87). This hypoxia imaging probe can show fast and stable MRI/fluorescence imaging signals, greatly improving imaging detection sensitivity.

#### 3.3.2 AQ4N

AQ4N, also known as banoxantrone, is a highly soluble di-N-oxide prodrug. It was designed to have minimal cytotoxicity in the presence of oxygen. Hypoxic tumor cells can activate and reduce it to a single N-oxide intermediate (AQ4M), which is ultimately reduced to the cytotoxic metabolite, AQ4 (112–114). It had been proved that the reduction of AQ4N into toxic AQ4 could be improved by further enhance the local hypoxia level of TME (115, 116). Coincidentally, PDT therapy could aggravate the hypoxia within tumor regions *via* continuous O2, so AQ4N can be used in combination with PDT. Zhang et al. constructed a tumor-specific nanoplatform (AQ4N-Cu(II)-Apt_Ce6_-GNP) using Cu(II)-liganded chlorin e6 (Ce6)-labeled aptamer-gold nanoparticles to host AQ4N. For this model, the TSL11a aptamer with tumor-targeting function is linked to AuNPs through Au-S bonds. After the particles are endocytosed by tumor cells, Au-S bonds are cleaved by a large amount of GSH in the cells and release Ce6 to enhance PDT. Compared with PDT (Ce6) or AQ4N treatment, the AQ4N-Cu(II)-Apt_Ce6_-GNPs group produced a more pronounced therapeutic effect after irradiation with a 670 nm laser. PDT aggravates tumor hypoxia, increases the amount of reductase, and enhances AQ4N activity, resulting in a superior synergistic antitumor effect (88). AQ4N combined with starvation therapy can also enhance the antitumor effect. Glucose oxidase (GOX) consumes glucose and oxygen to produce H_2_O_2_, which enhances hypoxia and oxidative stress in tumor cells. Liu et al. encapsulated AQ4N and GOX in long-circulating recessive liposomes, which effectively inhibited 4T1 tumor cells *in vivo* (117). Similarly, Yu and co-workers developed yolk–shell organosilica nanoparticles containing tetrasulfide bonds to deliver AQ4N and GOX. Increased intracellular GSH levels in tumor regions disrupt the tetrasulfide bond to release GOX, which subsequently consumes oxygen and glucose to produce H_2_O_2_. Further consumption of oxygen drives the conversion of AQ4N to toxic AQ4. Meanwhile, the depletion of GSH can further elevate the H_2_O_2_ levels. This combinatorial strategy had been proved by both *in vitro* and *in vivo* results (118). However, glucose is widely distributed in the human body, which means that the use of these two passive tumor-targeting nanocarriers involves high risk. Li et al. optimized this by choosing PEG and polycaprolactone (PCL) copolymers modified by peroxyoxalate (PO) (89). This nanocarrier is a vesicular structure that prevents drug leakage while in circulation. When PEG accumulates around the tumor, PO reacts with the large amount of H_2_O_2_ in the tumor area to enhance the permeability of the PEG membrane. The reaction between glucose oxidase and glucose entering the PEG promoted the production of H_2_O_2_. Finally, AQ4N is activated and produces cytotoxicity through cascade amplification. Importantly, in normal tissues, glucose oxidase cannot react with glucose because of blocking by the PO barrier structure, ensuring the safety of PEG.

#### 3.3.3 Azo

Azo compounds can be decomposed under low-oxygen conditions to generate luminescent amino derivatives; this unique property originates from azo bonds. The azo bond, with the structural formula –N=N–, undergoes reversible reductive cleavage in a normoxic environment (119). This process of converting non-luminescent azo compounds into luminescent products can be used to develop hypoxia small-molecule probes and hypoxia-triggered prodrugs while combining them with nanomaterials for better diagnostics and treatment effects. A hybrid liposome (HR-HLP), composed of azo and hydrogenated soybean phospholipids (HSPC), achieves this goal (120). Stimulating the reduction products to azo in the TME traps the HR-HLP in the tumor regions, and the ensuing carrier cleavage releases the loaded drug to produce antitumor effects. This process was monitored using NIR fluorescence imaging. Azo compounds can also be used to enhance PDT. In the hydroxyapatite nanosystem, azobenzene, a representative hypoxia-responsive compound, can link human serum albumin (HSA)-coated Ce6 chloride with oxaliplatin (HCHOA). The nanosystem can quickly dissociate into ultrasmall Ce6-conjugated HAS (HC) and oxaliplatin prodrug-conjugated HAS (HO) therapeutic nanoparticles with a diameter smaller than 10 nm under hypoxia TME. Owing to their ultra-small particle size, HC and HO therapeutic nanoparticles can easily penetrate the core of the tumor away from the blood vessels. At the same time, Ce6 has extremely low activity when coated with nanoparticles, but its fluorescence and singlet oxygen production abilities increase rapidly when it is released. Singlet oxygen could selective apoptosis induction in tumor cells. This special property gives the nanocomposite a lower imaging background signal and better light-induced efficacy (90). In addition, azobenzene improved PEGylation siRNA delivery. Early experiments found that azobenzene-linked PEG, PEI, and 1,2-dioleyl-sn-glycero-3-phosphoethanolamine (DOPE) nanocarrier complexes (PAPDs) could be activated by hypoxia and cleaved to isolate siRNA (121). PAPDs retain their stability in normal tissues, but they cannot effectively silence genes, indicating that parts of this nanostructure or siRNA can be improved. Huang et al. chose an iron (Fe)-azo metal-organic framework (AMOF) and adsorbed HIF-1α siRNA on its surface for targeted therapy (91). AMOF carriers have two advantages over the PAPDs. First, a positively charged metal frame can be better adsorbed onto the surface of the negatively charged cell membrane, promoting tumor cell endocytosis. Second, HIF-1α has a stronger inhibitory effect on tumor growth after silencing. The results demonstrated the unique advantages of AMOFs in hypoxia response activation *in vivo* and *in vitro*.

## 4 Discussion

**Figure 4:**
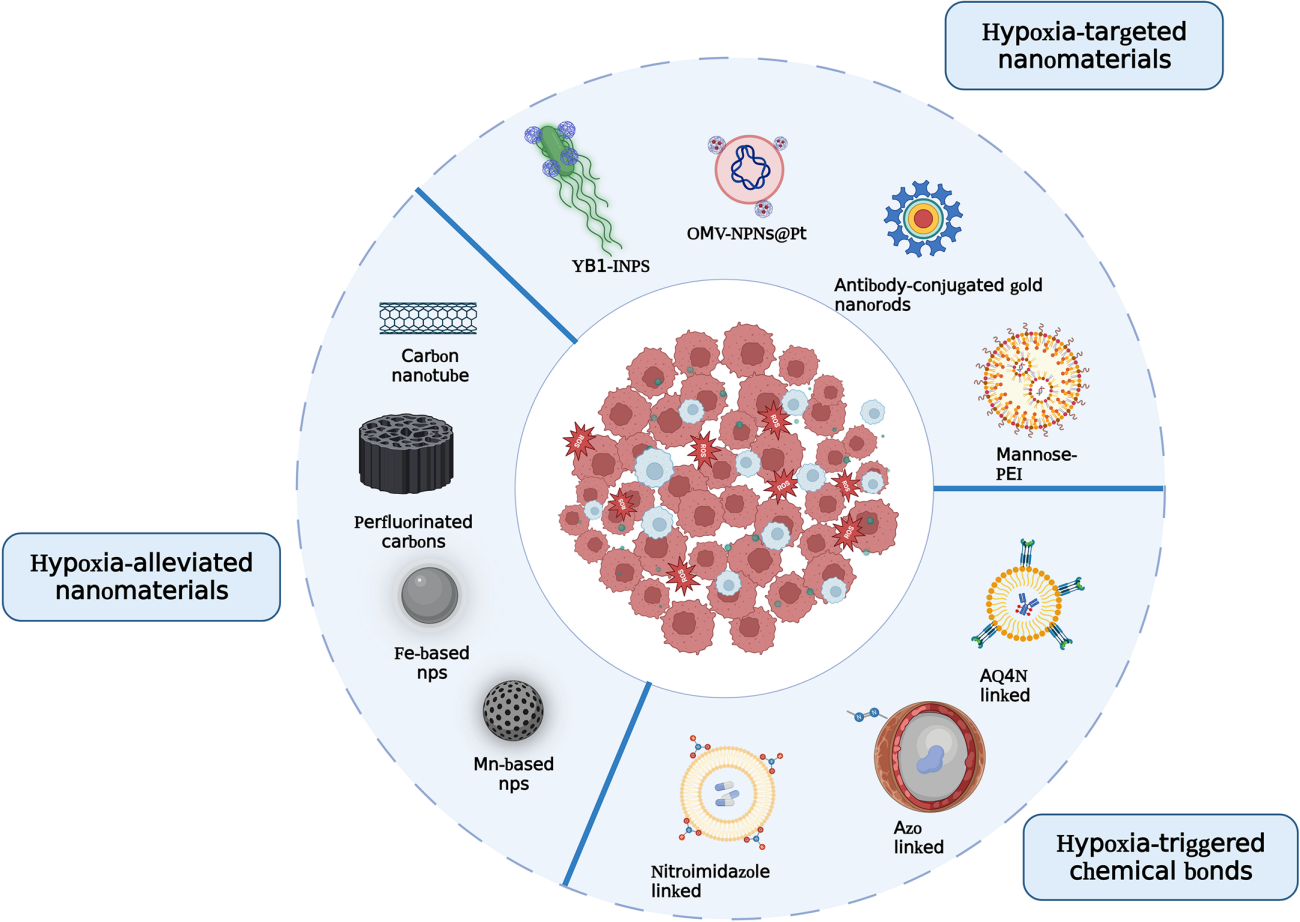
Illustration of the principles to design hypoxia-responsive nanomaterials.

Hypoxia-responsive nanomaterials can be used for diagnostics, therapeutics, or both. Previous attempts to target tumor hypoxia were based on the development of hypoxia-activated prodrugs or small-molecule inhibitors directed toward tumor cells. These prodrugs and inhibitors are usually difficult to be delivered to tumors because of the poor vasculature and high interstitial pressure in the TME. The transport of drugs to undesired locations or uncontrolled drug release may lead to an increase in adverse effects. Nanomaterials can retain drug concentrations for a longer duration by passively or actively accumulating in the tumor regions. In the present study, various nanoplatforms that release encapsulated drugs into the TME—bacteria-mediated hypoxia-specific nanoparticles, hypoxia-selective chemical bond-conjugated nanomaterials, and TAM-targeted nanocarriers—displayed favorable prospects as hypoxia-specific therapeutics (**Figure 4**). Their use can enrich the efficacy of chemotherapy, PDT, PTT, and other therapeutic approaches. Nano-contrast agents for MRI, PET, and NIR imaging, such as SPIONPs and mCMSNs, may provide more accurate and earlier tumor detection than existing contrast agents. In addition to the achievements of these nanoparticles, there are still some issues that need to be addressed before clinical translation.

The biocompatibility of nanomaterials, particularly metal nanomaterials, should be considered. Because metal nanomaterials are difficult to be excreted from the body, they may lead to undesired inflammation and increase the risk of cytotoxicity. The extent of damage varies depending on the nanomaterial type and structural and functional characteristics, all of which must be carefully evaluated *via* preclinical studies. The clearance rate of the nanomaterials is another issue need to be considered. The main metabolic organs are the kidney and liver (122). Some nanoparticles had low clearance rates, such as gold nanomaterials with diameters greater than 150 nm, which could still be detected *in vivo* one year after the *in vivo* injection. The current study found that surface functionalization (hydrophilic moieties such as PEG and PLGA) and the small size of nanoparticles (<50 nm) could be crucial in reducing undesirable uptake (123). For example, nanoparticles possessing self-decomposition functions, such as PO-modified PEG-PCL nanocarriers, folic acid-modified PEG sandwich complexes, and fluorocarbon chain-linked silica complexes, can be rapidly excreted by liver and kidney, which has a unique advantage in clinical transformation.

The development of simple and smart nanomedicines in future studies will be important for their clinical application. Responsive nanoparticles are currently evolving in a multi-modal manner. Multi-strategy synergistic therapeutic nanocarriers, such as AQ4N synergistic PDT, Gd@HbCe6-PEG, and multi-layer iron-based nanomaterials, have been used in many previous studies and have shown excellent efficacy. However, owing to their multi-layer structure and complex synthetic procedures, the clinical transformation of these materials is limited. The synthesis of nanoparticles should be simple and facile, and there should be a uniform standard. Many researchers are moving toward making their nanoparticles out of materials that have been generally regarded by the U.S. Food and Drug Administration (FDA) as being easily scalable, such as SPION, Mesoporous silicananoparticles (MSNs), and so on. Furthermore, bacteria-based microbial synthesis has many advantages for the synthesis of metal nanomaterials (MNMs). Bacteria are easier to isolate and cultivate due to natural evolution, which could be mass-produced in a short time at low cost. Therefore, bacteria could rapidly synthesize a wide range of MNMs, such as Au, Fe_3_O_4_, CdTe, and so on. Researchers may also choose the template methods to prepare nanoparticles, which simplify the synthesis and assembly steps of nanomaterials and is suitable for mass production.

Although existing nanomaterials are still far from clinical applications, we believe that benefiting from the advances of nanotechnology, intelligence responsive nanomaterials will improve the clinical cancer imaging and therapy.
